# Primary osseous chest wall sarcomas: Oncological and surgical outcomes in a large single-institution cohort of 100 patients

**DOI:** 10.1016/j.jbo.2026.100772

**Published:** 2026-06-01

**Authors:** B.P.H Oteman, R.E. Evenhuis, M. Fiocco, G.M. Shahin, R.J.P van der Wal

**Affiliations:** a Department of Orthopaedic Surgery, Leiden University Medical Center; b Mathematical Institute Leiden University; c Princess Máxima Center for pediatric oncology, Utrecht, Netherlands; d Department of Biomedical Data Science, Section medical statistics, Leiden University Medical Center; e Department of Cardiothoracic Surgery, Leiden University Medical Center

## Abstract

**Introduction:**

Primary osseous chest wall sarcomas are rare tumours with limited evidence guiding surgical management. This study aimed to evaluate oncological outcomes, complication rates, and prognostic factors associated with survival in a large, single-institution cohort.

**Methods:**

We retrospectively reviewed 100 patients who underwent chest wall resection for primary osseous sarcoma between 1994 and 2022. Outcomes included overall survival (OS), event-free survival (EFS), local recurrence (LR), distant metastasis (DM), and postoperative complications. The cumulative incidence of LR and DM were estimated using competing risk models. Prognostic factors were analysed with Cox regression.

**Results:**

The cohort included 79 patients with chondrosarcoma, eight with osteosarcoma, and 13 with Ewing sarcoma. Negative margins (R0) were achieved in 79%. Reconstruction was performed in 68%, most commonly with mesh. Postoperative complications occurred in 19%. Five-year OS was 79% (70–87%) for the entire cohort, and by subtype: osteosarcoma 38% (4–71%), Ewing sarcoma 67% (41–94%), and chondrosarcoma 85% (77–94%). Recurrence patterns differed, osteosarcoma showed a high 5-year risk of DM (75% (40–100%)), chondrosarcoma predominantly exhibited LR (15% (7–23%)), and Ewing sarcoma both LR (15% (0–36%)) and DM (40% (11–69%)). Notably, grade 1 chondrosarcoma showed metastatic potential, emphasizing that the risk of distant metastasis should also be considered in these low-grade tumours, with a 5-year cumulative incidence of distant metastasis was 11% (95% CI: 0–27%). Tumour size and residual tumour (R1/2) were associated with worse outcomes.

**Conclusions:**

Radical resection (R0) remains the most important predictor of survival. 5-year survival differs substantially by subtype (chondrosarcoma 85%, Ewing sarcoma 67% and osteosarcoma 38%), while tumour size is associated with a less favourable prognosis. Individualized reconstructive strategies enable complex resections with acceptable morbidity (19% postoperative complications).

## Highlights

• R0 resection is the strongest predictor of survival in chest wall sarcomas.
• 5-year survival differs by subtype: chondrosarcoma 85%, Ewing 67%, osteosarcoma 38%.
• Acceptable complication rate (19%) despite complex surgery.
• Tumour size >6 cm associated with worse oncological outcomes.

## 1 Introduction

Primary tumours of the chest wall are rare, and among them, osseous sarcomas represent a particularly uncommon subset. Sarcomas account for approximately 1% of all malignant tumours, and only 10–15% of these arise in the chest wall [1]. Among the primary chest wall tumours, roughly 55% originate from bone or cartilage, with the remaining 45% arising from soft tissue [2]. The most common malignant osseous tumours in this region include chondrosarcoma, osteosarcoma, and Ewing sarcoma, with chondrosarcoma being the most frequent [3].

Due to their rarity and heterogeneity, chest wall sarcomas pose diagnostic and therapeutic challenges. The literature consists largely of small retrospective cohort studies or case series, and standardized guidelines for optimal treatment are lacking. As a result, treatment strategies often depend on tumour histology, anatomical location, and institutional preferences. Complete surgical resection with negative margins remains the cornerstone of treatment for almost all patients with chest wall sarcomas [4]. Achieving negative margins is critical, whereas positive margins have been associated with increased rates of local recurrence and reduced disease-free survival [5], [6].

However, balancing oncological radicality with preservation of function and reduction of surgical morbidity remains challenging. Clear resection margins increase disease free survival, although a more radical resection is associated with increased risk of complications, including respiratory dysfunction, infection, and chest wall instability [7]. To restore chest wall stability, function, and cosmesis, complex reconstructions using synthetic or biological materials are often required, further increasing the risk of postoperative complications. Pulmonary complications (eg. pneumonia, pleural effusion, atelectasis, (persistent) pneumothorax, acute respiratory failure) have been reported in up to 30% of patients [8], and surgical site infections occur in up to 23% of patients [9]. Although higher infection rates have been reported in patients with large defects (>3 ribs) and in those receiving prosthetic material [10], it remains uncertain whether this increased risk is primarily related to defect size, the use of prosthetic material, a combination of both, or other contributing factors such as patient-related characteristics.

There is currently no guideline for the role of (neo)adjuvant therapies, such as chemotherapy and radiotherapy, in chest wall sarcomas, and their use remains controversial in selected cases. Their use is guided by tumour subtype, size, resectability and further individual patient characteristics [11]. Neoadjuvant radiotherapy may be considered in selected cases, especially in the context of preoperative anticipated positive margins, large tumour volume or unresectable disease, but evidence supporting its effectiveness is limited [11].

Long-term outcomes for patients with malignant osseous chest wall tumours remain poor. Five-year overall survival rates are estimated around 60–75%, while 10-year survival declines to approximately 45–60%, depending on histological subtype, margin status, and presence of distant metastases at diagnosis [12], [13], [14].

Given the rarity of primary osseous chest wall sarcomas and the limited evidence guiding their surgical and oncological management, we aimed to evaluate oncological outcomes including time to local recurrence (LR), distant metastasis (DM), and overall survival (OS) as well as complication rates and prognostic factors, in a large long term single institution cohort.

## 2 Methods

A retrospective cohort study of patients who underwent chest wall resection for a sarcoma between 1994 and 2022 at a tertiary oncological referral centre; Leiden University Medical Center (LUMC) was conducted. After ethical approval from the institutional ethic review committee, 227 consecutive chest wall resection specimens for sarcoma were identified in the histopathologic database (Delpic) of the department of Pathology of the LUMC.

**Fig. 1:**
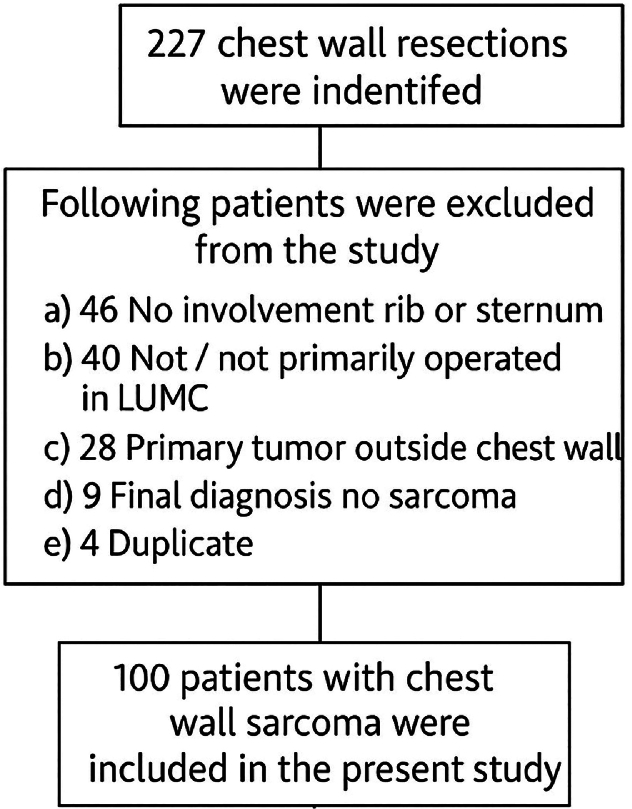
Flowchart.

Chest wall sarcomas were defined as any malignant sarcoma involving ribs, sternum and costovertebral junctions. Tumours arising from the scapula, clavicle, vertebra or soft tissue only were excluded, as these locations involve fundamentally different surgical approaches and the chest wall is often not primarily involved in their resection. Additionally, patients who underwent prior biopsy at an outside institution were included, whereas patients who underwent primary resection or who had prior incomplete resection at another institution were excluded. (Fig. 1). All patients were discussed in a multidisciplinary team meeting before surgical procedures were performed by specialized thoracic, neurosurgical oncologists, and orthopaedic oncologists.

### 2.1 Data collection

Data on patient demographics, tumour characteristics, surgical details, complications, and survival were collected from electronic patient records (HiX, ChipSoft, Amsterdam). The extent of resection (e.g., number of ribs, sternal involvement) and reconstructive techniques (e.g. use of surgical mesh, fascia lata allograft, and/or musculocutaneous flaps) were documented. The closest distance from tumour tissue to resection margin was collected as bone and soft tissue margin. However, due to anatomical borders of the chest wall (e.g., the pleura), soft tissue margins were often not recorded or considered meaningful and therefore were not included in the analyses. (Neo)-adjuvant treatments, including chemotherapy and/or radiotherapy, were recorded when applicable. Immunotherapy was not included, as it currently has no established role in these tumours, and none of the patients in our cohort received this treatment. We assessed response to the neoadjuvant chemotherapy with the Huvos classification [15]. Follow-up was calculated from the date of surgery until last date of follow-up or death. Postoperative complications were recorded for all patients and categorized as major or minor. Major complications were defined as events requiring surgical intervention or reoperation, while minor complications were managed conservatively with medical therapy, such as antibiotics for pneumonia and wound infections or transfusions.

### 2.2 Outcomes

The primary outcomes were overall survival (OS), defined as the time from surgery to death from any cause, and event-free survival (EFS), defined as the time from surgery to the first occurrence of LR, DM, or death for any cause. LR was defined as a relapse of primary tumour situated at the same location of the primary tumour which was radically or marginally resected. For patients with macroscopic residual disease (R2), who underwent surgery with palliative intent, progression at the operative site represents persistent residual disease rather than true postoperative recurrence. Secondary outcomes included complication rate, incidence and pattern of LR and DM and identification of prognostic factors associated with OS, LR and DM.

### 2.3 Statistical analysis

Categorical data was presented as contingency tables (frequencies and percentages). For continuous data, summary statistics of mean and standard deviation (SD) or median with interquartile range (IQR) was presented. OS and EFS from surgery were estimated using the Kaplan-Meier method. Patients alive at last follow-up were censored. The Log-rank test was used to test the difference in survival outcomes between groups. Median follow-up was estimated with reverse Kaplan–Meier [16]. To estimate the cumulative incidence of local recurrence (LR) and distant metastasis (DM), a competing risks model was employed with death as a competing event [17]. Difference between the cumulative incidence was assessed with the Gray's test [18]. To study the impact of prognostic factors on survival outcomes, Cox regression models were estimated. Due the limited number of events relative to the number of prognostic variables and based on the events-per-variable criterion (minimum 10 events per variable), only univariable regression models were estimated. Hazard ratios (HR) and 95% confidence intervals (CI) were reported for OS. Cause specific Hazard ratio (HR_CS_) along with 95% CI were reported for LR and DM. The proportional hazard assumptions were tested for the variables sex, tumour size, histological grade, bone margin, and residual tumour classification by using the weighted residuals [19]. To assess potential heterogeneity introduced by the study period, survival outcomes were compared between patients treated before and after 2010 by using log rank test for OS and EFS and Gray's test for the cumulative incidence of LR and DM.

To identify risk factors associated with postoperative complications, univariable logistic regression models were estimated. Sex, age, BMI, ASA score, tumour size, number of resected ribs, use of reconstruction, neoadjuvant chemotherapy, neoadjuvant radiotherapy, and residual tumour classification were used as independent factors. Results are presented as odds ratios (OR) with 95% confidence intervals.

All analyses concerning the competing risk model were performed with the library mstate [20] and the library cmprsk [21] in the R software environment [22]. The remaining statistical analysis were performed with IBM SPSS 29.

## 3 Results

### 3.1 Baseline characteristics

A total of 100 patients treated for primary bone sarcoma of the chest wall were included (Table 1). The cohort consisted of 60 males and 40 females, with a median follow up of 7.2 years (IQR (5.7–8.6), a mean age of 50 years and a mean BMI of 26.1. Two patients (2%) presented with metastatic disease at diagnosis. The most frequent diagnosis was chondrosarcoma (79%), followed by Ewing sarcoma (13%) and osteosarcoma (8%). Most tumours were limited to the ribs (54%), followed by combined rib and sternal (15%), rib and vertebrae (12%) and isolated sternal (8%) involvement. Extension into adjacent structures occurred in 7% (lung), 3% (clavicle), and 1% (diaphragm). Neoadjuvant chemotherapy was administered in 19% of patients, and neoadjuvant radiotherapy in 6%, while 16% and 9% received adjuvant chemotherapy and radiotherapy, respectively. All Ewing sarcoma patients received neoadjuvant chemotherapy. The use of (neo-)adjuvant therapies by histological subtype is presented in Table 2.

**Table 1:** Patient demographics.

Variable		N (%)	Mean (CI)
Gender	Female	40 (40%)	
	Male	60 (60%)	
BMI			26 (25–27)
Oncological diagnosis	Chondrosarcoma *Grade 1* *Grade 2* *Grade 3* *Dedifferentiated*	79 (79%) *23 (29%)* *40 (51%)* *11 (14%)* *5 (6%)*	
	Osteosarcoma	8 (8%)	
	Ewing sarcoma	13 (13%)	
ASA score	1	32 (32%)	
	2	57 (57%)	
	3	11 (11%)	
Age at surgery (yrs)	Chondrosarcoma	79 (79%)	53.95 (49.94–57.97)
	Osteosarcoma Ewing sarcoma	8 (8%) 13 (13%)	55.66 (32.92–78.39) 20.12 (13.52–26.71)
Max tumour diameter (cm)			6.6 (5.9–7.4)
Duration of surgery			3 h (2.5 h – 3.5 h)
Amount of bloodloss (ml)			1264 (308–2219)
Number of ribs involved	≤2 >2	59 (59%) 41 (41%)	
Metastatic disease at presentation		2 (2%)	
Anatomical involvement	Ribs only Ribs and sternum Sternum only Ribs and vertebrae Lung Clavicle Diaphragm	54 (54%) 15 (15%) 8 (8%) 12 (12%) 7 (7%) 3 (3%) 1 (1%)

**Table 2:** The use of (neo-)adjuvant therapies by histological subtype.

	Chondrosarcoma (*n* = 79)	Osteosarcoma (n = 8)	Ewing sarcoma (n = 13)	Total (*n* = 100)
Neoadjuvant chemotherapy	2 (3%)	4 (50%)	13 (100%)	19 (19%)
Neoadjuvant radiotherapy	1 (1%)	1 (13%)	4 (31%)	6 (6%)
Adjuvant chemotherapy	0 (0%)	5 (63%)	11 (85%)	16 (16%)
Adjuvant radiotherapy	4 (5%)	1 (13%)	4 (31%)	9 (9%)

Reconstruction was required in most cases. No reconstructive material was used in 32%, while 40% underwent reconstruction with a surgical mesh. Fascia lata allograft was used either alone 7% or in combination with a surgical mesh 5%. Additionally, 5% underwent reconstruction with a myocutaneous or pedicled flap, spinal instrumentation was placed in 9%, and polymethyl methacrylate (PMMA) was applied in 2 cases, both of whom underwent large sternal excisions. In one of these cases, PMMA reconstruction was combined with a myocutaneous flap. No bone allografts or any metal reconstructions were used. The choice of reconstructive technique was guided by the number of resected ribs and anatomical location of the defect, as detailed in Table 3. Single rib resections were most managed without reconstruction (21/32, 66%) or with mesh alone (8/32, 25%), whereas resections of two or more ribs consistently required structural reconstruction in most cases. Fascia lata, either alone or in combination with mesh, was used predominantly in larger defects involving three or more ribs. Multiple rib resections (>1 rib) consistently required structural reconstruction, except when defects were caudal (allowing coverage by adjacent musculature) or cranial/dorsal near the spine (where intrinsic muscle coverage was often sufficient). In one case (>1 rib) without structural reconstruction and with the defect located outside regions typically covered by muscle a lung herniation occurred. These findings emphasize tailoring reconstruction strategies to defect location and extent.

**Table 3:** Reconstruction method by number of resected ribs.

Resection	None	Mesh	Fascia lata	Fascia lata + Mesh	Flap	Instrumentation	PMMA
Sternum only	1	0	1	0	1	0	0
1 rib	21	8	1	0	0	2	0
2 ribs	5	14	2	0	1	2	0
3 ribs	5	14	2	5	1	3	0
4 ribs	0	3	1	0	1	1	0
5 ribs	0	1	0	0	0	1	0
6 ribs	0	0	0	0	0	0	2

### 3.2 Margins

Following surgical resection, microscopically negative margins (R0) were achieved in 79% of patients, whereas 13% had microscopically positive margins (R1), and 4% had macroscopic residual tumour (R2). All 4 R2 cases were preoperatively anticipated and performed with palliative intent (debulking procedure). Mean distance from the tumour margin to osseous resection margin (bone margin) was 16 mm (95% CI: 13–19). Bone margin of <1 cm was not associated with higher risk of LR or DM with a HR_cs_ of 1.5 (95% CI: 0.3–6.8) and 1.3 (95% CI: 0.4–4.9) respectively (Table 4).

**Table 4:** Hazard ratio (HR) and 95% confidence interval (CI)for prognostic factors estimated from a univariable Cox regression model of prognostic factors for overall survival (OS), local recurrence (LR), and distant metastasis (DM) in patients with chondrosarcoma (n = 79). For LR and DM outcomes cause-specific hazard ratio (HRCS) and 95%CI are reported.

	OS: HR (95% CI)	P	LR: HR_CS_ (95% CI)	P	DM: HR_CS_ (95% CI)	P
Sex						
Female						
Male	1.2(0.4–3.3)	0.78	2.0 (0.6–6.5)	0.23	2.7(0.7–9.7)	0.14
Histological grade						
I & II						
III	10.1(2.8–35.8)	<0.01	2.8(0.8–10.5)	0.12	2.8(0.7–10.9)	0.14
Size						
Per cm increase	1.1 (0.9–1.2)	0.42	1.1(1.0–1.3)	0.02	1.3 (1.1–1.4)	<0.01
Residual tumour classification						
R0						
R1&R2	3.3(1.2–9.2)	0.02	3.5 (1.2–10.1)	0.02	2.9(1.0–9.0)	0.06
Bone margin						
<1 cm						
>1 cm	0.3(0.1–1.6)	0.16	1.5 (0.3–6.8)	0.58	1.3(0.4–4.9)	0.68

### 3.3 Complications

Postoperative complications occurred in 19 patients (19%) of whom 8 (8%) needed reoperations. The overall complication rate consisted of 8% major complications (requiring reoperation) and 11% minor complications (managed conservatively). The most frequent complications included pleural effusion (*n* = 5, 5%), wound infection (*n* = 3, 3%), excessive bleeding (n = 3, 3%), pneumonia (*n* = 2, 2%), reconstructive failure (n = 2, 2%), pulmonary embolism (*n* = 1, 1%) and pericarditis (n = 1, 1%). All cases of pleural effusion were strictly surgery-related and not associated with disease progression, local recurrence or metastatic involvement. Among the eight patients who underwent reoperation, four were treated for pleural effusion, three by video-assisted thoracoscopic surgery (VATS) and one underwent debridement and mesh removal. Two patients required re-exploration due to wound infection and excessive bleeding. One patient with progressive kyphoscoliosis underwent discectomy from Th8–Th12 due to reconstructive failure, and one pneumothorax was managed with thoracic drain insertion. BMI was the only variable significantly associated with postoperative complications (OR 1.12, 95% CI: 1.01–1.24, *p* = 0.031).

### 3.4 Oncological outcomes

**Fig. 2:**
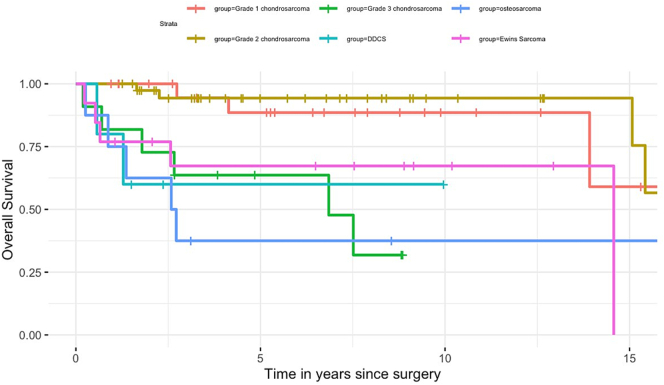
Estimated overall survival (OS) per diagnosis

**Fig. 3:**
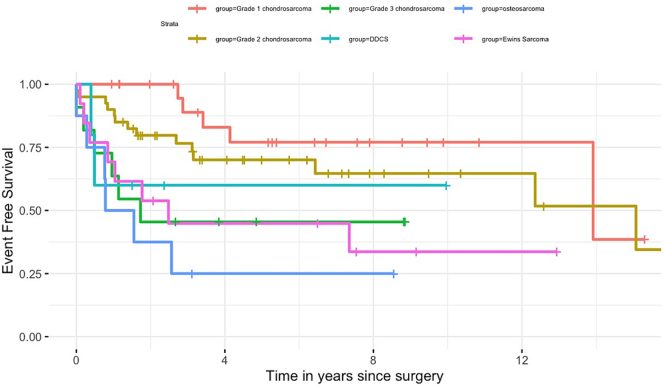
Estimated Event Free Survival (EFS) per diagnosis

During follow-up, 18 patients (18%) had developed LR, and 26 patients (26%) had developed DM. The distribution of metastatic sites is presented in Table 5. A total of 25 patients had died by the end of the follow-up period. Median follow-up time was 7.2 years (IQR: 5.7–8.6). Overall, 5-year survival for the entire cohort was 79% (95% CI:70–87%). At 5 years, OS was 38% (95% CI:4–71%) for osteosarcoma, 67% (95% CI:41–94%) for Ewing sarcoma, and 85% (95% CI:77–94%) for chondrosarcoma (Fig. 2). By histological grade chondrosarcoma had a 5-year OS of 89% (95% CI: 74–100%) for grade I, 94% (95% CI: 87–100%) for grade II, and 64% (95% CI: 35–92%) for grade III. The 5-year event-free survival for the entire cohort was 61% (95% CI: 51–71%). By diagnosis, 5-year EFS was 67% (95% CI: 56–78%) for chondrosarcoma. Chondrosarcoma EFS for grade was 77% (95% CI: 57–97%), 70% (95% CI: 54–85%) and 46% (95% CI: 16–75%) for grade 1, 2 and 3 respectively. 5-year EFS for Ewing sarcoma was 45% (95% CI: 17–73%) and 25% (95% CI: 0–55%) for osteosarcoma (Fig. 3).

**Table 5:** Oncological outcomes.

Variable		N (%)	Mean (CI)
Deceased		25 (25%)	
Local recurrence		18 (18%)	
Distant Metastasis	Overall	26 (26%)	
	Lung	17 (17%)	
	Bone	9 (9%)	
	Soft tissue	6 (6%)	
	Liver	2 (2%)	
	Brain	1 (1%)	
Complication	No	81 (81%)	
	Yes *N requiring reoperation (major)*	19 (19%) *8 (42%)*	
Residual tumour classification	R0	79 (79%)	
	R1	13 (13%)	
	R2	4 (4%)	
Histological response (Huvos)	I (0–49% necrosis)	5 (5%)	
	II (50–89% necrosis)	4 (4%)	
	III (90–99% necrosis)	5 (5%)	
	IV (100% necrosis)	2 (2%)

**Fig. 4:**
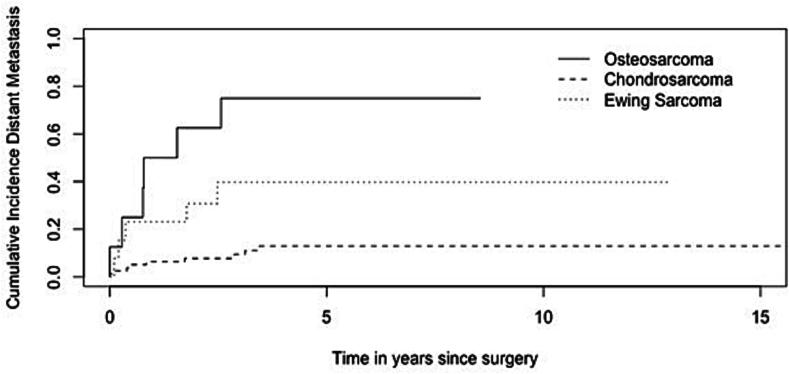
Cumulative incidence of distant metastasis (CIDM) for diagnosis

**Fig. 5:**
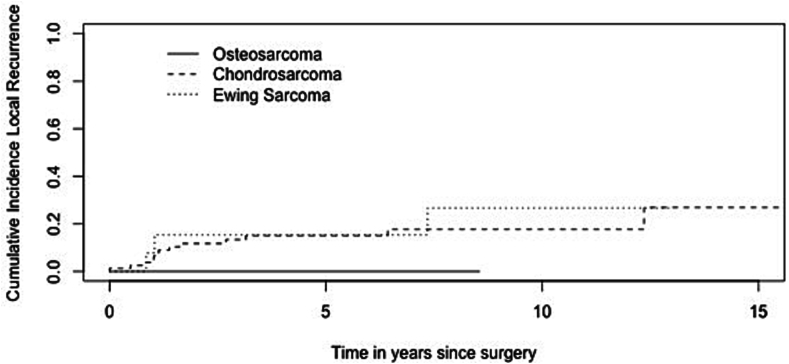
Cumulative incidence of local recurrence (CILR) for diagnosis

**Fig. 6:**
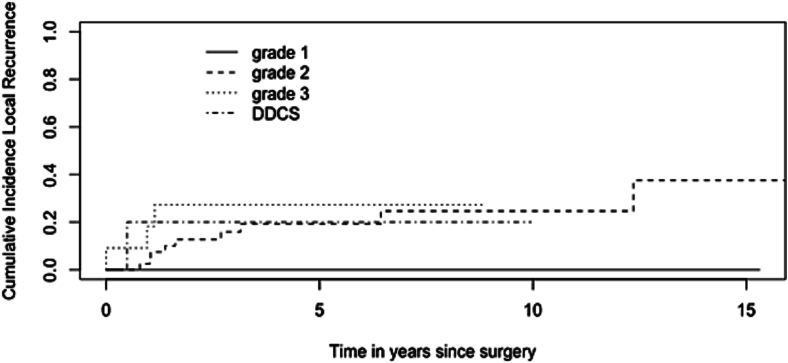
Cumulative incidence of local recurrence (CILR) for Chondrosarcoma patients according to different grades and dedifferentiated chondrosarcoma (DDCS).

**Fig. 7:**
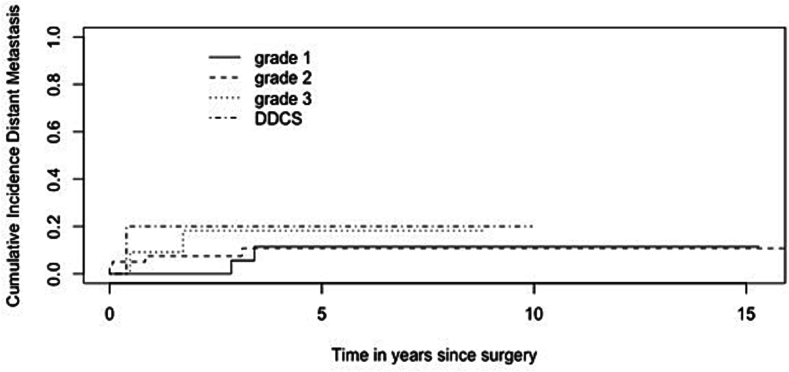
Cumulative incidence of distant metastasis (CIDM) for Chondrosarcoma patients according to different grades and dedifferentiated chondrosarcoma (DDCS).

Cumulative incidence of local recurrence (LR) and distant metastasis (DM) for the different histological subtypes are shown in Fig. 4, Fig. 5, Fig. 6, Fig. 7. No cases of LR occurred in osteosarcoma patients within 5 years after surgery, whereas both chondrosarcoma and Ewing sarcoma showed a cumulative incidence at 5 years of 15% for LR (95% CI 7–23% and 0–36%, respectively). Regarding DM, osteosarcoma showed the highest cumulative incidence, reaching 75% (95% CI 40–100%) at 5 years, followed by Ewing sarcoma with 40% (95% CI 11–69%) and chondrosarcoma with 13% (95% CI 5–21%). When stratified by grade among chondrosarcoma patients, no significant differences were observed between subtypes. The 5-year cumulative incidence of LR was 0% for grade 1, 19% (95% CI 6–33%) for grade 2, 27% (95% CI 0–55%) for grade 3, and 20% (95% CI 0–60%) for dedifferentiated chondrosarcoma (DDCS). The corresponding 5-year cumulative incidence of DM was 11% (95% CI 0–27%) for grade 1, 11% (95% CI 1–21%) for grade 2, 18% (95% CI 0–43%) for grade 3, and 20% (95% CI 0–59%) for DDCS.

### 3.5 Prognostic factors

Univariate Cox regression model on 79 chondrosarcoma patients identified tumour size and residual tumour classification (R1/2) as significantly associated with worse OS, LR and DM. Hazard ratios and cause-specific hazard ratio along with the 95% confidence intervals are presented in Table 4. In chondrosarcoma patients,tumour size was not significantly associated with OS (HR 1.1, 95% CI: 0.9–1.2, *p* = 0.415), but was significantly associated with LR (HRcs 1.1, 95% CI: 1.0–1.3, *p* = 0.018) and DM (HRcs 1.3, 95% CI: 1.1–1.4, *p* = 0.001). Histological grade (grade 3 vs grade 1 and 2 combined) was significantly associated only with OS (HR: 10.1(95% CI 2.8–35.8), but not with LR or DM. The proportional hazard assumption was not violated.

## 4 Discussion

This study estimated a 5-year overall survival of 85% (95% CI: 77–94%) for chest wall chondrosarcoma which is consistent with the higher end of published ranges, which vary widely from 60% to over 90% depending on the specific cohort and reporting period [[1], [2], [4], [13], [23]]. However, the survival varied per grade with a 5-year OS of 89% (95% CI: 74–100%) for grade I, 94% (95% CI: 87–100%) for grade II, and 64% (95% CI: 35–92%) for grade III. This supports the need for radical resection regardless of histological grade. Although our results show only minor differences between grade I and II, other studies have reported more pronounced prognostic differences between low- and intermediate-grade tumours [4], [14]. In the chondrosarcoma subgroup, histological grade was associated with LR but not with DM. Tumour size was associated with higher hazard for LR and DM. While our analysis showed no statistically significant association between bone margin width (>1 cm) and LR, DM or OS in our cohort, these findings should be interpreted with caution given the limited sample size. Achieving the widest possible margin remains oncological desirable, and R0 resection remains a critical determinant of outcome. These results underscore the importance of achieving R0 margins even in complex locations, despite the histological grade, as this remains the strongest modifiable predictor of outcome.

Different recurrence patterns were observed across the histological subtypes. Osteosarcoma patients rarely developed local recurrence but showed a high cumulative incidence in distant metastases, reaching 75% (95% CI: 40%–100%) within 5 years.None of the osteosarcoma patients had a history of prior radiotherapy, indicating that no radiation-associated osteosarcomas were present in our cohort. In contrast, chondrosarcoma patients primarily experienced LR of 15% (95% CI: 7–23%) with relatively low rates of DM of 13% (95% CI: 5–21%) at five years. Ewing sarcoma experienced LR of 15% (95% CI: 0–36%) and a substantial risk of DM of 40% (95% CI 11–69%) at 5 years. These differences suggest that follow-up protocols should be tailored by histology in chest wall sarcomas, where systemic surveillance remains essential for all chest wall sarcomas.

An important finding of our study was that grade 1 chondrosarcomas still showed metastatic potential, with a cumulative incidence of 11% (95% CI 0–27%). This contrasts with previous research on appendicular tumours, which reported no metastases in low-grade cases [24]. As the chest wall is part of the axial skeleton, our observed metastasis rates are consistent with current evidence, underscoring the importance of adequate resection for all grades of chondrosarcoma in the chest wall [24].

A notable finding was that 68% of patients required prosthetic or flap reconstruction, most commonly mesh. Multiple rib resections (two or more ribs) consistently required structural reconstruction, except when defects were cranial or caudal and could be covered by intrinsic musculature. The single case of lung herniation occurred when prosthetic support was not used outside regions typically covered by muscle. This emphasizes that reconstruction strategies should be tailored to defect location and number of ribs resected. Despite the high proportion of complex resections involving sternum, spine, or lung, postoperative complication rate remained 19% which is at the lower end of published ranges (15–30%) [3], [4], [8], [14]. This favourable outcome may in part be attributed to our multidisciplinary surgical approach, patient-tailored reconstructions, and extensive institutional experience with these rare tumours. BMI was the only significant predictor of postoperative complications.

Analysing soft tissue margins in chest wall resection cohorts is particularly challenging. Due to anatomical constraints, such as proximity to the pleura and lungs, quantifying these margins in millimetres is often difficult or even meaningless. Tumours can be completely resected without invasion of adjacent structures, yet reported margins may be ≤1 mm or not specified. This reflects an inherent aspect of chest wall surgery and should be considered in the design and interpretation of future studies.

Histological response to neoadjuvant chemotherapy (Huvos score) is a well-established prognostic factor for survival and risk of local recurrence or distant metastasis, particularly in Ewing sarcoma and osteosarcoma [25], [26]. Given that most patients in our cohort had chondrosarcoma, which is not treated with chemotherapy, the Huvos score was only applicable to a subset of patients (*n* = 16). For these patients, findings are presented descriptively, which is appropriate given the histological composition of this cohort.

Our study has several limitations inherent to its retrospective single-centre design. The study relies on the accuracy of documentation made for clinical and not for research purposes. This introduces the risk of information bias due to potential inaccuracies or missing details that were deemed clinically irrelevant. Secondly, collected data covered nearly three decades (1994–2022) during which diagnostic and therapeutic approaches might have evolved. To address potential heterogeneity introduced by the nearly three-decade study period, a subgroup analysis comparing outcomes between patients treated before and after 2010 was performed. No statistically significant differences were observed in OS (*p* = 0.264), EFS (*p* = 0.781), LR (*p* = 0.825), or DM (*p* = 0.685) between the two periods. It should be noted that follow-up time is shorter for patients treated after 2010, which may limit the interpretation of long-term outcomes in this subgroup. Thirdly, given the rarity of osteosarcoma and Ewing sarcoma of the chest wall, sample sizes for these subtypes were small (*n* = 8 and *n* = 13, respectively), which is reflected in the wide confidence intervals in their presented outcomes.

To the best of our knowledge, this single-institution study of 100 patients represents the largest retrospective cohort focused exclusively on primary osseous chest wall sarcomas and contributes meaningful data to the literature field of this rare disease. This study offers valuable insights into the surgical management of chest wall sarcomas over a prolonged period.

## 5 Conclusion

Our findings highlight four central messages:

• Radical resection (R0) remains the strongest modifiable predictor of survival.
• Tumour size is a critical prognostic factor and underlines the importance of early referral to specialized centres.
• Survival outcomes differ considerably between tumour types, with chondrosarcoma exhibiting the most favourable prognosis.
• Complex chest wall reconstructions can be performed with acceptable morbidity, provided reconstruction strategies are tailored to the anatomical defect and location.

These results add to the limited literature on primary osseous chest wall sarcomas and support an aggressive but carefully individualized surgical approach to optimize long-term outcomes.

## CRediT authorship contribution statement

**BPH Oteman:** Conceptualization, Data curation, Formal analysis, Investigation, Methodology, Visualization, Writing-original draft, Writing-review & editing. **R.E. Evenhuis:** Conceptualization, Data curation, Resources, Writing-review & editing. **M. Fiocco:** Formal analysis, Methodology, Validation, Visualization, Writing-review & editing. **G.M. Shahin:** Resources, Validation, Writing-review & editing. **R. J.P. van der Wal:** Supervision, Conceptualization, Data curation, Formal analysis, Methodology, Project administration, Writing-review & editing

## Declaration of competing interest

The authors declare that they have no known competing financial interests or personal relationships that could have appeared to influence the work reported in this paper.
